# Tertiary hyperparathyroidism in patients with pseudohypoparathyroidism type 1a

**DOI:** 10.1016/j.bonr.2022.101569

**Published:** 2022-04-14

**Authors:** Masatsune Itoh, Michiko Okajima, Yuko Kittaka, Akihiro Yachie, Taizo Wada, Yutaka Saikawa

**Affiliations:** a Department of Pediatrics, Kanazawa Medical University, Kanazawa, Japan; b Department of Pediatrics, School of Medicine, Institute of Medical, Pharmaceutical and Health Science, Kanazawa University, Kanazawa, Japan; c Department of Pediatrics, JA Onomichi General Hospital, Onomichi, Japan

**Keywords:** PHP, pseudohypoparathyroidism, PHP1a, pseudohypoparathyroidism type 1a, PHP1b, pseudohypoparathyroidism type 1b, PHP1c, pseudohypoparathyroidism type 1c, PTH, parathyroid hormone, HPT, hyperparathyroidism, Gsα, Gs-alpha, AHO, Albright's hereditary osteodystrophy, TSH, thyroid stimulating hormone, GHRH, growth-hormone-releasing hormone, C-PTH, carboxyl terminal fragments of PTH, BMI, body mass index, BMD, bone mineral density, HBS, hungry bone syndrome, PHP1a, Pseudohypoparathyroidism 1a, Tertiary hyperparathyroidism

## Abstract

Pseudohypoparathyroidism type 1a (PHP1a) is a genetic disorder caused by heterozygous loss-of-function mutations on the maternal allele of the *GNAS* gene. Patients with PHP1a predominantly exhibit parathyroid hormone (PTH) resistance and physical features of Albright's hereditary osteodystrophy. We report two unrelated cases with PHP1a who developed tertiary hyperparathyroidism (HPT). Molecular analyses of the *GNAS* gene identified a previously known heterozygous 4-bp deletion (c. 565_568delGACT) in exon 7 in case 1 and a novel heterozygous missense mutation (p.Lys233Glu) in exon 9 in case 2. Both patients developed tertiary HPT associated with hyperfunctioning parathyroid glands during long-term treatment of hypocalcemia. Case 1 had severe osteoporosis and underwent parathyroidectomy. Case 2 was asymptomatic with no evidence of bone diseases associated with tertiary HPT. PHP1a patients are at risk of developing tertiary HPT and should be treated with sufficient doses of calcium and vitamin D to achieve serum PTH levels within the mid - normal to double the upper limit of the normal range, regardless of serum calcium levels.

## Highlights

• Pseudohypoparathyroidism type 1a induces tertiary hyperparathyroidism.
• Tertiary hyperparathyroidism can be complicated by hungry bone syndrome.
• Serum PTH levels should be within doubled the upper limit of normal.

## 1 Introduction

Pseudohypoparathyroidism type 1 (PHP1) is a rare genetic disorder, in which affected patients manifest hypocalcemia and hyperphosphatemia despite elevated serum parathyroid hormone (PTH) levels. PHP1 is further classified into three types according to the presence (PHP1a, OMIM 103580 and PHP1c, OMIM 612462), or absence (PHP1b, OMIM 603233) of physical features, termed Albright's hereditary osteodystrophy (AHO), which include short stature, obesity, round face, subcutaneous ossification, brachydactyly, and other skeletal anomalies (Mantovani, 2011; Mantovani and Spada, 2006; Turan and Bastepe, 2015). PHP1a is caused by heterozygous inactivating mutations within the maternal *GNAS* gene, leading to insufficient expression of the Gs-alpha (Gsα) subunit. Gsα is required for membrane signal transduction by hormones such as PTH, thyroid stimulating hormone (TSH), gonadotropins, and growth-hormone-releasing hormone (GHRH), whose target tissues show predominant expression of Gsα from the maternal *GNAS* allele. Consequently, PHP1a patients develop resistance to these hormones of variable severity during childhood or adolescence. PHP1b consists of isolated renal resistance to PTH with lack of typical AHO features. PHP1b patients have imprinting/methylation defects at the *GNAS* locus, accounting for loss of maternal Gsα protein expression in the renal tissue. PHP1c is clinically identical to PHP1a in terms of the presence of AHO and hormone resistance, except for normal erythrocyte Gs activity. This form is considered to be a variant of PHP1a (Mantovani et al., 2018).

Recently, the European Consortium for the study of PHP (EuroPHP) network proposed the term “inactivating PTH/PTHrP signaling disorder (iPPSD)” as a new classification instead of pseudohypoparathyroidism, in which they classified disorders of impairment of the PTH/PTHrP signaling pathway based on the pathophysiology. Accordingly, PHP1a, PHP1c, pseudopseudohypoparathyroidism, and progressive osseous heteroplasia are classified as iPPSD2 due to loss of function mutation in GSα, and PHP1b is named iPPSD3 and occurs due to methylation changes at the *GNAS* differentially methylated region (Thiele et al., 2016).

Tertiary hyperparathyroidism (HPT) is a rare condition, in which patients develop autonomous parathyroid hyperfunction leading to persistent hypercalcemia and elevated serum PTH. This is often the result of long-standing secondary HPT, occurring most commonly in patients with chronic kidney disease (Evenepoel, 2013; Gioviale et al., 2012), although all PHP1 (PHP1a and PHP1b) patients are theoretically at risk of developing tertiary HPT. Persistent HPT leads to progressive bone diseases, osteitis fibrosa cystica, and soft-tissue calcification. Although tertiary HPT is classically caused by hyperplasia of all four glands, over 20% of patients might have single or double adenomas as the underlying pathology (Bizzarri et al., 2016). Surgical parathyroidectomy is indicated in patients with clinical symptoms of tertiary HPT, such as fatigue, pruritus, bone pain, non-traumatic fracture, nephrocalcinosis, and peptic ulcer disease (Tang et al., 2017). Previously, only five cases of PHP1b who developed tertiary HPT have been reported (Neary et al., 2012), and no cases of tertiary HPT following PHP1a have been reported.

We herein describe two cases of PHP1a who developed tertiary HPT during long-term treatment for hypocalcemia.

Consent was obtained from the patients for publication of their case reports and any accompanying images.

## 2 Case reports

### 2.1 Case 1

A 32-year-old male first presented with abdominal osteoma cutis at the age of 7 months, although with no abnormalities in serum calcium levels and renal function. At the age of 4 years, he experienced a generalized seizure associated with severe hypocalcemia (5.8 mg/dL, normal range 8.7–10.3 mg/dL) and high levels of serum carboxyl terminal fragments of PTH (C-PTH) (1.0 ng/mL, normal range < 0.5 ng/mL). He had no known family history of hypocalcemia. An Ellsworth-Howard test revealed the presence of PTH resistance (Table 1), and physical examination showed AHO features of short stature (−2.8 SD), obesity (body mass index, BMI 21.6), round face, subcutaneous ossification and brachydactyly.

**Table 1:** Preoperative and most recent post-operative data in PHP1a patients.

Case	1	2
PTH infusion test (Ellsworth-Howard test)		
Urinary excretion of phosphorus (PHP < 35 mg/2 h)	−15.1	20.3
Urinary excretion of cAMP (U4-U3) (PHP < 1 μmol)	−3.8	0.016
Urinary excretion of cAMP (U4/U3) (PHP < 10)	1.0	1.7

Preoperative		
Serum calcium (8.5–10.3 mg/dL)	11.9	10.1
Serum phosphorus (2.5–4.7 mg/dL)	3.4	3.5
Alkaline phosphatase (115–359 U/L)	15,375	569
Intact PTH (10–65 pg/mL)	6720	2112
Urinary calcium/creatinine ratio (< 0.21 mg/mg)	0.32	0.15
Bone mineral density (T-score of the lumbar spine)	−3.9	−0.4

Postoperative (with concomitant medications)		
Years since surgery	6	Observation
Serum calcium (8.5–10.3 mg/dL)	9.8	
Serum phosphorus (2.5–4.7 mg/dL)	3.6	
Alkaline phosphatase (115–359 U/L)	393	
Intact PTH (10–65 pg/mL)	22	
Urinary calcium/creatinine ratio (<0.21 mg/mg)	0.08	
Bone mineral density (T-score of the lumbar spine)	−1.7	
Alphacalcidol (μg/d)	0.5	

He was clinically diagnosed as PHP1a and treatment with oral calcium lactate (2.0 g/day) and alphacalcidol (1.5 μg/day) was started. Levothyroxine was added for the treatment of non-autoimmune hypothyroidism that developed at the age of 9 years. From the age of 26 to 32 years, while he was under treatment with calcium lactate (0–2.0 g/day) and alphacalcidol (0.5–2 μg /day), a gradual increase in serum intact PTH levels was observed, with a corresponding increase in serum alkaline phosphatase (ALP) levels, despite normalization of serum calcium and phosphorus levels. His mean (range) serum intact PTH levels, serum ALP, urinary calcium excretion (urinary calcium/creatinine ratio), serum calcium, and serum phosphorus levels before the diagnosis of tertiary HPT were 3108.9 pg/mL (1350–4590 pg/mL, normal range 10–65 pg/mL), 745.0 U/L (143–5175 U/L, normal range 115–359 U/L), 0.18 mg/mg (0.09–0.28 mg/mg, normal range < 0.21 mg/mg), 9.93 mg/dL (9.4–10.3 mg/dL), and 3.19 mg/dL (2–2.4 mg/dL), respectively.

**Fig. 1:**
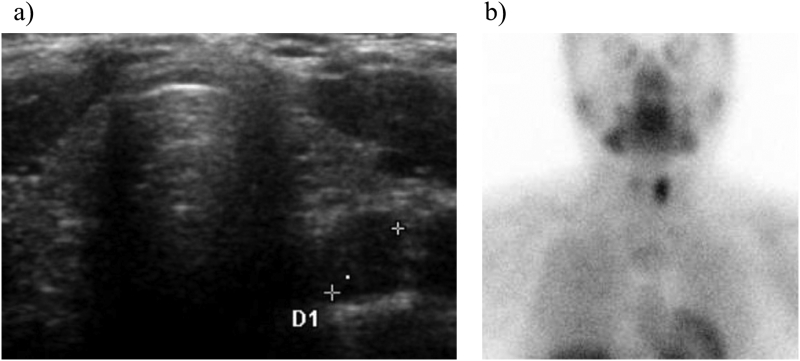
a) In case 1, ultrasonography revealed a 3.53 × 1.65 × 1.28 cm solid mass localized in the right inferior thyroid gland. b): ^99m^Tc-MIBI scintigraphy identified a hyperfunctioning right inferior parathyroid gland in case 1.

At the age of 32 years, he presented with hypercalcemia (11 mg/dL) with extremely high serum intact PTH (4640 pg/mL) and ALP (5983 U/L) levels, although with normal serum phosphorus levels (3.1 mg/dL). Imaging studies revealed an enlarged hyperfunctioning right inferior parathyroid gland (Fig. 1a, b), and bone mineral density (BMD) evaluations indicted severe osteoporosis (0.496 g/cm^2^, T-score of −3.9 SD at the lumbar spine), consistent with a diagnosis of tertiary HPT. Thereafter, he sustained a spontaneous fracture of the left proximal humerus, and computed tomography examination showed multiple brown tumors at the site of pain in the right tibia and fibula caused by tertiary HPT.

The patient subsequently underwent parathyroidectomy, and histopathologic evaluation of the parathyroid gland (3.5 × 2.5 × 1.3 cm) showed the presence of an adenoma. Intraoperative PTH monitoring showed a drop in PTH levels from 7525 to 345 pg/mL after removal of the parathyroid gland, and postoperative PTH levels were normalized at 11 months after surgery. Shortly after parathyroidectomy, he developed hypocalcemia (4.7–8.4 mg/dL) associated with hypophosphatemia (2.0–2.4 mg/dL, normal range 2.5–4.7 mg/dL) and hypomagnesemia (1.5–1.7 mg/dL, normal range 1.8–2.4 mg/dL), attributed to “hungry bone syndrome” (HBS). He required long-term treatment for HBS with administration of high doses of calcium and alphacalcidol to maintain adequate serum calcium and PTH levels. The lumbar spine T-score values increased from −3.9 to −1.7 at 9 months after surgery. At the time of 6 years after surgery, he was under treatment with a reduced dose of alphacalcidol (0.5 μg/day), and continued to maintain normocalcemia (9.8 mg/dL) and, normophosphatemia (3.6 mg/dL), and normal levels of serum intact PTH (22 pg/mL), serum ALP (393 U/L), and urinary calcium/creatinine ratio (0.08 mg/mg) without symptoms, and his lumbar spine T-score values were maintained at −1.7 SD. Furthermore, his left humerus fracture healed without delay, and brown tumors gradually improved.

### 2.2 Case 2

**Fig. 2:**
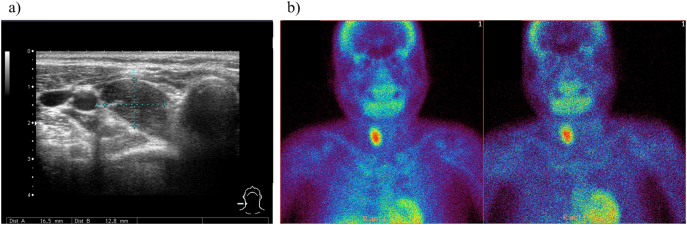
a) In case 2, ultrasonography revealed an 8 mm-solid mass indicated as D1 localized in the left inferior thyroid gland. b) ^99m^Tc-MIBI scintigraphy identified a hyperfunctioning left inferior parathyroid gland in case 2.

A 25-year-old female first presented with a generalized seizure associated with hypocalcemia (2.1 mg/dL), hyperphosphatemia (10.5 mg/dL), and elevated serum intact PTH levels (524.8 pg/mL) at the age of 4 years. The patient had no known family history of hypocalcemia. She had PTH resistance on the Ellsworth-Howard test (Table 1), non-autoimmune hypothyroidism, and AHO features of short stature (−3.1 SD), obesity (BMI 22.6), round face, short neck and brachydactyly. She was clinically diagnosed as PHP1a and treatment with oral calcium, alphacalcidol and levothyroxine was started. From the age of 7 to 15 years, she required a high dose of alphacalcidol (2.5 μg/day) since her serum intact PTH levels occasionally increased above 400 pg/mL despite normalization of serum calcium levels. Before the diagnosis of tertiary HPT, her mean (range) serum intact PTH, serum ALP, urinary calcium/creatinine ratio, serum calcium, and serum phosphorus levels during her clinical course were 451.5 pg/mL (47.2–1549 pg/mL), 362.1 U/L (132–569 U/L), 0.04 mg/mg (0.01–0.15 mg/mg), 8.74 mg/dL (6.6–10.0 mg/dL), and 5.62 mg/dL (4.0–9.0 mg/dL), respectively. She experienced menarche at the age of 14 years. At 17 years, her serum calcium level exceeded 10 mg/dL (10.1 mg/dL), corresponding to a rise in serum intact PTH levels from 379 to 510 pg/mL. Despite reduction of alphacalcidol doses from 2.5 μg/day to 2 μg/day, her serum calcium levels remained above 10 mg/dL and intact PTH levels increased further from 567.5 to 1507 pg/mL. Imaging studies revealed a hyperfunctioning left inferior parathyroid gland (Fig. 2a, b), consistent with a diagnosis of tertiary HPT. Since she was asymptomatic and had no BMD-based evidence of osteoporosis (1.076 g/cm^2^, T-score of −0.4 SD at the lumbar spine), she was conservatively managed with medical monitoring alone and remains asymptomatic more than 8 years later. Her latest reports showed serum intact PTH, serum ALP, urinary calcium/creatinine ratio, serum calcium, and serum phosphorus levels were 573 pg/mL, 170 U/L, 0.01 mg/mg, 9.5 mg/dL, and 4.0 mg/dL, respectively. Further, she did not develop overt hyperparathyroid bone disease, and her recent lumbar spine T-score values were −0.3 SD.

## 3 Mutation analysis of the *GNAS* gene

Each of the patients provided informed consent for mutational analysis. The ethics committee of Kanazawa Medical University approved this study. The patients' genomic DNA was extracted from their white blood cells. The 13 coding exons and intron-exon boundaries of the *GNAS* gene (Ensembl identifiers: gene ENSG00000087460; transcript, ENST00000265620.11) were analyzed for mutations, as previously described (Mantovani et al., 2000). An identified missense mutation in case 2 was evaluated for pathogenic potential using three mutation tolerance prediction approaches (multiple *in silico* analyses): PolyPhen-2 (http://genetics.bwh.harvard.edu/pph2/) (Adzhubei et al., 2010), SIFT (http://sift.jcvi.org) (Ng and Henikoff, 2001), and Mutation Taster (http://www.mutationtaster.org) (Schwarz et al., 2014). DNA testing of the patients' parents was not performed in both cases.

## 4 Results

**Fig. 3:**
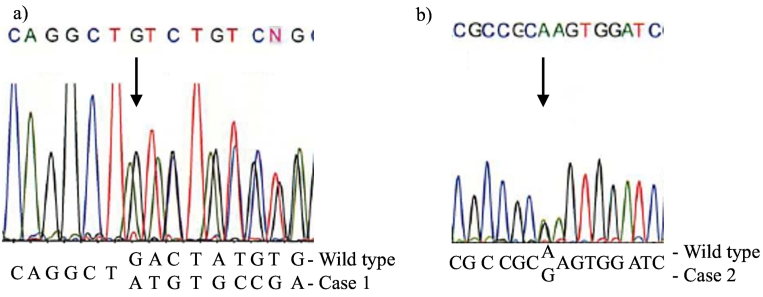
a) In case 1, the patient had a 4 bp deletion in one allele of exon 7 (p.Asp189Met*14) (thin arrow). b) In case 2, the patient had an adenine to guanine transition (A > G) in codon 697, resulting in a substitution of lysine by glutamic acid (p.Lys233Glu) (thick arrow).

In case 1, sequencing of the entire coding region of the *GNAS* gene revealed a heterozygous 4-bp deletion mutation at position 565 (c. 565_568delGACT) in exon 7 (Fig. 3a). This mutation resulted in a frameshift and premature termination (p.Asp189Metfs*14), as previously reported (Ahrens et al., 2001; Weinstein et al., 1992).

In case 2, a heterozygous missense mutation, c.697A > G (p.Lys233Glu), was newly identified in exon 9 (Fig. 3b).

**Fig. 4:**
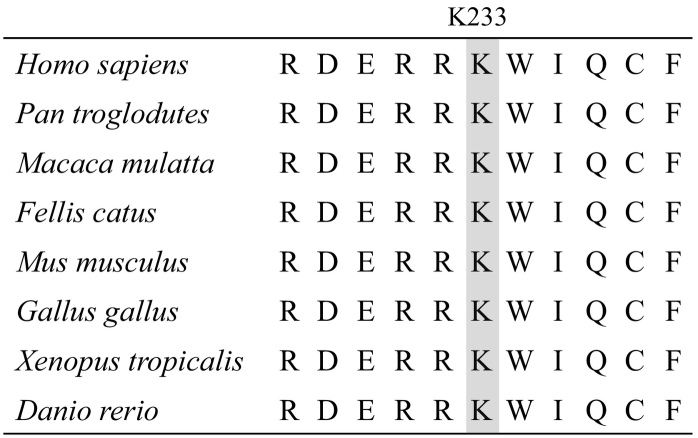
Evolution conservation of the lysine residue at the 233rd codon (www.ensemble.org). The conserved lysine is highlighted in the shaded box.

This single nucleotide substitution has not been found in the gnomAD (https://gnomad.broadinstitute.org/), iJGVD (https://jmorp.megabank.tohoku.ac.jp/ijgvd/), ClinVar (https://www.ncbi.nlm.nih.gov/clinvar/) or The Human Gene Mutation Database (http://www.hgmd.cf.ac.uk/ac/index.php). The Lys-233 residue in the Gsα protein is strictly conserved across various species (Fig. 4).

Two independent *in silico* analyses predicted the pathogenicity of this amino acid substitution with high probability scores: “probably damaging” (score of 0.993) by PolyPhen-2, and “damaging” (SIFT score = 0) by SIFT.

## 5 Discussion

We described two PHP1a patients who developed tertiary HPT associated with excessive secretion of PTH from hyperfunctioning parathyroid glands after an interval after diagnosis of 28 years in case 1 and 13 years in case 2. An essential component of the diagnosis of tertiary HPT is that continuous stimulation of the parathyroid glands by long-standing secondary HPT leads to unresponsiveness to negative feedback mechanisms (Tang et al., 2017). As in our patients, long intervals in the development of tertiary HPT were previously also reported in five PHP1b patients with tertiary HPT that occurred at a median interval of 34 years after the diagnosis of PHP1b (Neary et al., 2012). It is of interest that all PHP1 patients are theoretically at risk of developing tertiary HPT, although its occurrence has, to date, only been described in patients with PHP1b (Neary et al., 2012). The patients in our report improved symptomatically following treatment with calcium and an active vitamin D analogue (alphacalcidol), which normalized serum calcium and phosphorus levels, although they eventually developed hypercalcemia in association with progressive increase in serum PTH levels. It is likely that most patients with PHP1 who receive therapy to increase serum calcium levels experience relief of symptoms, but the therapeutic doses are often not high enough to normalize PTH levels (Neary et al., 2012).

Surgical intervention with parathyroidectomy is the most effective cure in patients with tertiary HPT. Although evidence-based guidelines on the selection criteria for parathyroidectomy in tertiary HPT patients are still inconsistent, the following indications for surgery have been proposed (Pitt et al., 2009): presence of severe hypercalcemia (serum calcium >11.5–12 mg/dL), persistent hypercalcemia (serum calcium >10.2 mg/dL for more than 3 months to one year), severe osteopenia (low bone mineral density), or symptomatic HPT (fatigue, pruritus, bone pain or pathologic bone fractures, peptic ulcer disease, mental status changes, or history of renal calculi).

In case 1, the patient had persistent hypercalcemia with severe osteoporosis, and, hence, was indicated for surgery. After parathyroidectomy, he developed postoperative HBS, which is a relatively uncommon, but serious adverse effect after parathyroidectomy. HBS develops when serum PTH levels decrease suddenly, such as following parathyroidectomy for hyperparathyroidism, with a shift in bone metabolism from one of resorption to net formation, and an influx of minerals into the bone leading to lowering of serum calcium and phosphate levels (Cartwright and Anastasopoulou, 2022). Risk factors for HBS include elevated levels of PTH, ALP, BMI, blood urea nitrogen, large size of removed parathyroid glands, and bone disease associated with HPT, such as bone fractures. In particular, patients with secondary HPT often demonstrate an increase in their serum PTH levels from 700 to 1000 pg/mL, and HBS is more likely to occur in patients with secondary HPT compared to primary (Jain and Reilly, 2017). In our case 1, he had many risk factors for the development of HBS, since both secondary and tertiary HPT have similar pathophysiology in terms of PTH resistance. The primary treatment of HBS is restoration of depleted skeletal calcium, although there are no clear guidelines for the management or prevention of HBS to date (Witteveen et al., 2013).

Some studies have also recommended the use of bisphosphonates or vitamin D supplementation for the prevention of HBS (Jain and Reilly, 2017). In case 2, the patient's serum calcium levels were below 10.2 mg/dL at the time of diagnosis of tertiary HPT, and she was asymptomatic with no evidence of bone diseases associated with tertiary HPT. With her consent, she was conservatively managed with medical monitoring of serum calcium, ALP and PTH levels, urinary calcium levels and BMD, without the use of calcimimetics.

Hypocalcemia associated with PHP is treated with active vitamin D metabolites or analogues and oral calcium supplements. However, these treatments to correct serum calcium levels suppress serum PTH levels, and can lead to the development hypercalciuria and renal calcification (Mantovani et al., 2018). Therefore, serum PTH levels should be maintained within the mid - normal to double the upper limit of normal range to reduce the risk of developing autonomous parathyroid and tertiary HPT, regardless of serum calcium levels (Mantovani et al., 2020). This recommendation is consistent with the clinical course of our two cases.

A positive molecular test helps to confirm the clinical diagnosis and to categorize the patient into a specific subtype of PHP, which provides important information for the medical management of the patient. The heterozygous 4-bp deletion mutation (c.del565_568) in exon 7 seen in case 1 is frequently found in PHP1a patients (up to 20% of reported mutations), representing a mutational hot spot (Lemos and Thakker, 2015). Mutations can be either maternally inherited or occur *de novo*, with both types of mutations having similar incidences (Elli et al., 2016). A missense mutation, c.697A > G (p.Lys233Glu), was newly identified in exon 9 of the *GNAS* gene in case 2. Previously reported mutations in exon 9 are missense mutations, including c.682C > T (p.Arg228Cys), c.691C > T (p.Arg231Cys), c.692G > A (p.Arg231His) (Human mutation 36:11–19, 2015), c.662 T > C (p.Met221Thr), c.701G > A (p.Trp234X), and c.713 T > G (p.Phe238Cys) (Thiele et al., 2015). These known mutations at codon 228 through 238 are all involved in the GTP dependent conformational change domain encoded by exon 9 (Fig. 1c). Functional studies (Farfel et al., 1996) for c.692G > A (p.Arg231His) demonstrated that the mutation led to a disturbance of the interaction between the switch 2 and 3 regions of Gsα, which are necessary to stabilize the active conformation. Taken together with the predictions by *in silico* analyses, the missense mutation at codon 233 identified in case 2 might have impaired Gsα function by reducing the stability of active conformation of the GTP dependent domain.

In conclusion, tertiary HPT can occur as a serious complication of PHP1 (PHP1a and 1b) when the therapeutic doses of vitamin D and calcium are insufficient to normalize PTH levels. Patients with tertiary HPT and persistent hypercalcemia and hyperparathyroid bone disease are indicated for surgical parathyroidectomy. Prospective studies are needed for optimizing pre- and postoperative treatment strategies in patients at a high risk for HBS, in order to prevent postoperative HBS.

## Funding

This research did not receive any specific grant from funding agencies in the public, commercial, or not-for-profit sectors.

## CRediT authorship contribution statement

Study design: MI and YS.

Patients' sample and clinical data collection: MO and YK.

Study conduct: MI, MO, YK, AY, TW and YS.

Data analysis: MI.

Data interpretation: MI and YS.

Drafting of manuscript: MI.

Revising manuscript: MI and YS.

MI and YS take responsibility for the integrity of the data analysis.

## Declaration of competing interest

None.
